# Honouring complexity and social justice in mental health: The socio-political economy of global mental health framework

**DOI:** 10.1371/journal.pmen.0000648

**Published:** 2026-08-04

**Authors:** Rochelle A. Burgess, Sorcha Ní Chobhthaigh, Bijayalaxmi Biswal, Valentina Iemmi, Diana Ceccolini, Babatunde Fadipe, Crick Lund, Soumitra Pathare

**Affiliations:** 1 Institute for Global Health, UCL, London, United Kingdom; 2 Department Social Work and Community Development, University of Johannesburg, Johannesburg, South Africa; 3 Division of Psychiatry, University College London, London, United Kingdom; 4 School of Health and Social Care, University of Essex, Colchester, United Kingdom; 5 London School of Hygiene & Tropical Medicine, London, United Kingdom; 6 Leicestershire Partnership NHS Trust, Leicester, United Kingdom; 7 Centre for Global Mental Health, Health Service and Population Research Department, Institute of Psychiatry, Psychology and Neuroscience, King’s College London, London, United Kingdom; 8 Department of Psychiatry and Mental Health, Alan J Flisher Centre for Public Mental Health, University of Cape Town, Cape Town, South Africa; 9 Centre for Mental Health, Law & Policy, Indian Law Society, Pune, Maharashtra, India; PLOS: Public Library of Science, UNITED KINGDOM OF GREAT BRITAIN AND NORTHERN IRELAND

## Abstract

Despite advances in acknowledging the importance of community, relational and family systems, and social adversities for mental health, responses in research and policy are limited. This calls us to explore methods to reconceptualize social dynamics as they relate to mental health outcomes. In this paper, we map out the potential of one framework, which directs attention to the domains associated with the social determination of poor mental health, the socio-political economy of global mental health. In this essay, we outline the need for such a framework, the theoretical underpinnings that inform it, as well as the potential social domains for inclusion. We conclude by outlining the potential implications of such a framework in shaping new practice in global mental health that is oriented towards a broader social justice lens – beyond emphasis on redistributive approaches, and towards collective and collaborative action to change the conditions that make poor mental health a reality for many – as part and parcel of our care systems.

*“And yet, the world has a choice: to honor or not to honor. The toll this choice takes has very real implications on our rights, our wealth, our justice” CA Riley, Black Liturgies [* 1 *].*

## 1 Background

Attention to the social in mental health has shifted from the margins to mainstream discourse. Yet even in the face of advancements in acknowledging the importance of adversity, community, relational and family systems within mental health, responses to these dynamics within mental health systems and services are limited. As a result, the depoliticization of social worlds linked to mental health has also been mainstreamed, such that some aspects of the social – such as family relationships, and culture – become readily acceptable as within the bounds of mental-health action, and others – such as the structural factors driving oppression and exclusion including political landscapes, are relegated to the space of prevention. This partial engagement has narrowed our perspectives on ‘social justice’ in the mental health landscape. The focus currently is primarily on the distribution of, and access to, services, as it relates to upholding the human right to health. This frequently overlooks discussion of where inequalities come from, and acknowledgement that they are inflicted on others along historical lines of exclusion and oppression within and between countries [2].

Critically, discussions of social justice in relation to mental health, must acknowledge the problematic framings that have limited our view and approaches to action. For example, across the mental health field, accounting for the social roots of conditions within the conceptualization and treatment of mental health problems has been a consistent focus of debate. Foucault’s notions of psychiatrisation- the mechanism through which psychiatry creates subjectivities and the boundaries of personhood aligned to state structures and desire of control [3]; or David Ingelby’s perspectives on critical psychiatry, which articulates the consequences of interpretation of societal violence and state failures as psychiatric illness [4] are two examples. This builds on early anti-colonial psychiatric perspectives championed by Frantz Fanon who also queried overly medicalized approaches to mental illness and clearly linked the impacts of the structural violence of colonialism and racism to experiences of distress and madness, which demanded treatments not of the mind, but the world which drives minds to madness [5]. Recent scholarship from Heidi Rimke [6] reiterates the importance of these early critiques, highlighting victim blaming that follows the erasure of social complexity within approaches to mental ill-health when they minimize state responsibility to build environments that enable good mental health. This legacy of thought demands alternative ways of embracing social conditions within framings of treatment and action.

In light of the above, it is also crucial to remember that social justice efforts, while incredibly diverse in their definitions and claims (see [7] for a useful reflection) can also fall prey to misuse. Typically, this occurs when action to address the roots of the unfair, avoidable differences in people’s lives resulting from oppression, exclusion, corruption, or other gross misuses or unequal distribution of power in the world is not centred [8,9]. Thus, we define social justice in mental health, as efforts to improve mental health that includes parallel active work to dismantle the systems driving unequal distributions of risk, as well as those driving unequal distributions of care. In such an approach, social justice is as much about questioning who has access to treatment, and why others don’t, as it is about amplifying efforts equalise access to the opportunities that make good mental health possible. Here we align with the Nancy Fraser’s articulation of social justice as it relates to the principles of participatory parity – or the factors drive the inability to participate politically and socially on equal grounds with peers [10]. Crucially, despite being anchored to western traditions, Fraser’s work resonates with decolonial scholarship on the problem of justice, including Thomas Sankara [11] revolutionary leader from Burkina Faso, whose visions for a socially just society acknowledged the multiple intersecting systems of oppression that drove limited social participation of his peoples: class, gender, contemporary manifestation of colonialism within political systems. Only through action on these issues, could claims for justice linked to health, housing, education – what we now deem the social determinants –be achieved.

Our attempts to achieve this brand of social justice within mental health is mandatory, but faces.many barriers, particularly in relation to treatment. One example emerges in how we define the social. While approaches to mental health causality have expanded to include key social and structural factors, we remain largely focused on what these factors produce for individual cases (the mental health condition), and less on what it means to live through these structural conditions within communities and broader societies. As a result, articulations of care and responses to social contexts centre on individual psychological consequences faced downstream, while mechanisms for managing the social relegates work on structural drivers into the landscape of ‘prevention’ (see [9]), with treatment work focusing more socio-relationally, leaving engagement with structural and political drivers outside of mental health systems. This has knock-on effects on how social justice issues are typically understood by practitioners within the mental health landscape, such that they focus on distributive justice (i.e., service inequalities), arguing for fair and more equitable distribution of access to services within society. This is necessary, but insufficient work. As argued in Iris Marion Young’s classic work on justice [12], conceptions and practices of social justice should begin with understanding *and acting* on the concepts of oppression and domination. Such a lens provides a platform for asking the critical and often underappreciated question within social determinants to mental health approaches: What are the factors and processes which *determine* the experience of negative social determinants and their impacts on mental health? This also resonates with work by scholars such as Sugerman [13] who argues that a lack of clarity on social justice framings, leads to a problematic permeability. This enmeshes with powerful neoliberal logics in mental health and psy-disciplines, establishing an emphasis on individualised care that ignores, or minimises related experiences of social and structural injustice. The result is that care infrastructures and interventions are not appropriately primed to respond to the complex experiences of oppression faced by patients and communities around the world.

In this essay, we argue that meaningfully addressing social determinants of mental health demands not just attention to inequalities, but also a deeper interrogation of the pathways that lead to mental ill-health, namely the complex experiences of oppression, discrimination and exclusion within wider social, political, and historical contexts of oppression and social disadvantage. We argue that the key factors which continually expose particular populations to the structurally embedded social determinants of poor health through history, can be better understood, and as a result, acted upon in practice, through a socio-political economy of global mental health. In this paper, we outline a framework for a *Socio-Politicalal Mentalb Economy of Glo Health* to center the social and structural determinants that yield poor mental health outcomes, through experiences of discrimination and exclusion, linked to a range domains that mark our current social, political and economic order.

### 1.1 Interconnected social realities: The socio-political-economic drivers of poor mental health

The social determinants of (mental) health approach has supported an increasing awareness of the ‘social’, and its relationship to biomedical and biological processes shaping experiences of mental ill-health [9]. However, this has not always resulted in action on the driving forces of social dynamics. For example, action on poverty is widely acknowledged to be a critical step in achieving mental health equity and the achievement of social justice in the long term [14]. However, poverty does not operate in people’s lives within a vacuum. Its impacts are complex and connected to the presence or absence of key social, relational, political, legal, and environmental phenomena, which have direct implications for not only the development of mental health difficulties, but also the success of their management. A closer engagement with the mechanisms that work to predispose certain populations to the perpetual and compounded experience of poverty is required. This helps us to interrogate the complex pattern of structural inequalities that both generate poverty and directly impact mental health. It draws attention to the oppressive systems that create and maintain intergenerational disadvantage – systems that are maintained by structural oppression.

For example, the presence or absence of poverty within Black South African communities, is directly related to the consequences of a history of apartheid political and economic structures, and the legacies that such structures have established within contemporary communities (see [15]). During apartheid, the introduction of a segregation policy formalising homelands for the Black population led to decreasing education attainment, employment rates, and earnings in affected children [16]. These political realities and the social structures they produced then further intersect with other *processes* in ways that influence patterns of poor mental health. Experiences of Black sex workers across contexts in South Africa [17] or Xenophobic attacks faced by Black Africans from elsewhere on the continent [18] highlight distinct experiences of risk associated with different social positions, impacting mental health outcomes. In the United States, examples of differential experiences of poverty within LGBT communities and Black LGBTQ+ communities (see [19]) as well as within disabled communities [20] illuminate the necessity of understanding where deprivation comes from to respond appropriately.

Many argue that poverty is a political choice [21] highlighting the ways in which particular policy landscapes create and embed poverty systematically among particular groups of people, across generations. There is a tendency to overlook how poverty is embedded within histories of exploitation and the workings of imperialism at both global and local levels. For example, discussions of farmer suicides in India clearly connect this phenomenon to geopolitical dynamics around seed sovereignty, state apparatuses which drive increasing debt burdens for famers [22], as well as intersections of caste discrimination [23]. Mental health support programming arguing for increased screening access or health education in such settings emerges as simultaneously helpful and harmful when geopolitical landscapes driving distress are ignored. In making sense of racially shaped health inequalities in the UK is often discussed in relation to the social gradient. A closer look at these spaces reveals the interwoven nature of multiple domains of oppression, where inequalities are also driven by intersecting political, class and colonial dynamics. For example, in south London where health inequalities are stark [24] historical shifts in neighborhoods like Brixton, including its most recent experiences of gentrification, are connected through time to multiple mechanisms of oppression that established and maintain today’s contemporary health inequalities. Despite the establishment of the neighbourhood by elite classes in the late 1800s, following the second world war bombardments and property damages drove elite classes out, and dwellings shifted from single owner, to tenement/multi-occupant homes, occupied by lower social classes. Post- World War 2 reconstruction also ushered in waves of Black and Irish migration, who were permitted to access housing in these areas given their lower desirability and quality [25]. Lack of investment over subsequent decades, has in recent years been described as degeneration ushered in under the guise of regeneration policies [26]. Any shifts in neighborhood dynamics and opportunities created by regeneration are rarely equally distributed, and access to improvements remains influenced by the intersections of racism and political dynamics, including over policing and the impacts of securitization narratives on Black communities in these areas [26].

The tendency to turn away from these complexities in our discussion of, and response to mental health inequalities, is not only unhelpful, but actively harmful, as it gaslights communities and generations into thinking such issues are in the past, and ‘*they should be beyond it’* arguments which underappreciates the impact of time within processes of change.

The reality is, that injustice and oppression are part of our recent and everyday histories and define our current social fabric. The end of Jim crow policies occurred only in the 1960s; Sweden’s forced sterilisation program continued until 1975; the South African formal apartheid system only ended in 1994 – the same year Finland criminalised marital rape; Canada’s last residential school closed in 1998 [27], the Windrush scandal re-traumatised Black Caribbeans in the UK this past decade [28], homosexuality remains criminalised in nearly 70 countries [29] and we continue to witness ICJ-labeled genocides in real time [30]. These realities simultaneously undermine communities’ economic health and sovereignty, which we then medicalise as individual pathology, while remaining active threats to the mental health of all individuals who access, provide and shape organisations of care.

As such, how we understand this risk and develop programmes of care must not only recognise these realities but consider and engage with the cumulative effects of multiple socio-political risk factors. This ultimately requires the centring of structural systems of power, and attending to broader social, political and economic challenges, structures and processes. Thus, to understand and appropriately respond to a downstream social determinant such as poverty requires an analysis of its source: the intersection of multiple social locations, and axes of oppression, both historical (e.g., apartheid economic policy and slavery) and contemporary (e.g., discrimination based on race, gender, work-roles, and policy landscapes fuelling xenophobic attitudes).

By anchoring particular social determinants of mental ill-health within the wider social, economic, and political structures of our world, we deepen our understandings of how they emerge and in turn, inform priorities for action in mental health policy and intervention planning [31] that resonate more strongly with people’s realities. Frameworks that make explicit both drivers of oppression in society and their intersections facilitate this process. Though not automatically praxis oriented, socio-political economies typically offer a framework that spotlights the mechanisms through which many common social determinants – such as poverty, or poor housing - come to impact some groups of people within societies more than others, including discrimination, marginalisation, and oppression. Scholars such as Nancy Kreiger, Ted Shreker and Jamie Breilh draw attention to how health and the causes of disease in the context of their social, political, and economic framings, as well as the contemporary and historical forces that produce the patterns of living and social conditions through which people’s health is determined [see: 32–34]. Such an approach is critical, as it views the factors that place health at risk as active, dynamic processes that reproduce patterns of poor health within and across generations, overcoming a structural blindness that has been linked to the social determinants approach, which acknowledges the political, but separates it from how we understand and how we engage with them as part of our practices of care. To take a step further, requires an emphasis on how health is *determined* within, and by social contexts, or ‘social determination’.

This crucial and alternative approach, *social determination*, is rooted within Latin American scholarship and praxis around social medicine and collective health. Jamie Breilh’s discussions of social determination draw attention to *how* things become [35]. Rooted in Marxist traditions, social determination theory is described by Harvey, Piñones-Rivera and Holmes [36] as linked largely to the principle of subsumption, – or rather how more complex social processes constrain and dominate a situation to create new ones. In the context of health; they are the factors that establish the limits and boundaries for action within particular domains of life. Breilh was interested in three domains: *general, particular and individual.* The most complex social processes exist within the general domain (i.e., broad structural dynamics and the social formations and mechanisms of their reproduction). The *particular* domain includes modes of living among groups with shared social categories, often organized across social hierarchies established by the general domain. Finally, at the individual level, styles of living form the focus – defined as the ability for an individual to organise their resources in ways that promote good health, drawing attention to formal memberships in organizations, identity, beliefs and values, and environmental settings. When combined, this framework embeds individuals within a network of social relations that continually draw our attention upward and outward – towards the general and specific dynamics that structure our social lives, and s the mechanisms through which we understand and navigate them.

The value of a social determination approach, is its ability to ensure that we carry the complexity of the social world with us in our theorising and planning for action. In making space for complexity – social determination in many ways, demands making explicit common aspects of the upstream socio-structural world (i.e., the general domain) that are relevant to, and typically overlooked within mental health spaces. In doing so, we make complexity more accessible, facilitating practitioners and policy actors in acknowledgment of the social factors that drive mental health needs in their totality. Social determinants, while critically important, on their own, are insufficient for coming to terms with the full range of mechanisms through which oppression operates across different communities and contexts. As argued by Harvey and colleagues [36] - social determinants approaches are limited by their interest in documenting inequality, as opposed to *equity* – which results in mainstream approaches that underestimates the role of power in the establishment of health outcomes. Specifically, Harvey and colleagues articulate that the separation of critical variables (such as income) from a holistic understanding of society, results in:

*“...reasonable responses that redirect attention (and sometimes responsibility and blame) to the actions of individuals and all fail to grasp the fundamental process of social reproduction underlying this tip of the iceberg empirically observed epidemiological fact”* [36]

Finally, intrinsic to the power of our proposed framework is the concept of intersectionality. In earlier work, we articulate the importance of intersectionality as an orientation to the frameworks’ application, which we reiterate here:

*“By articulating an intersectional approach to socio-political economy, we seek to direct actors to hold onto the complexity of people’s experiences, to enable awareness of, and conversations across differing levels of power (i.e., the interpersonal, structural, disciplinary and hegemonic domains) at work in people’s lives. As such, our framework depicts how structural determinants work as factors of oppression creating continual interactions, amplifying and creating unique experiences of risk of poor mental health among individuals and communities. Or more simply put,* ***promoting the understanding that acknowledging that risk is everywhere, is not the same as understanding the specific risks that factors pose for communities****.”* [2, emphasis added]

In this essay, we expand on why an intersectionality approach is mandatory to advancing social justice principles in global mental health. Intersectionality has begun to occupy a broader space in mental health. For example, Fassiner and colleagues [37] recent special issue on intersectionality in mental health practice, highlighting its role in ethics, clinical consultation and as a reflection tool. In most instances, these articulations emphasize the ability for varied social locations, to establish individual experiences of mental health. Their discussion, however, focuses on identity positions, often separate from the social conditions that establish them.

For example, explorations of discrimination within mental health prioritise relationships to the experience of discrimination stemming from the mental health condition itself; and more recently, acknowledging that this can co-occur with different forms of stigmatisation, including ethnicity or identity categories [38]. In many respects, when the emphasis is on mental health stigma and social exclusion of people living with mental health conditions, we often assume that the illness identity is the primary category through which oppression is mediated. As a result, we may develop incomplete understandings of how multiple social systems continually create and maintain mental health inequities through oppression by overlooking how social systems establish intersecting forms of stigma. Social justice approaches in mental health also center identity politics – organising around shared identity categories – rather than promotion of efforts to address the varied types of structural oppression which cut across multiple identity concerns [39]. The consequence is that we address individual symptoms of oppression (a stigmatised identity) rather than dismantling their social, political and economic structural antecedents [40].

This type of structural blindness becomes particularly problematic when we fail to acknowledge how political arenas not only shape the risk factors communities are exposed to, but also affect the mental health of those providing care, as well as the safety of organisations that deliver these services. For example, in contexts of open-ended trauma or violence, it is rarely acknowledged that service users and providers may often be navigating the same type adverse socio-political circumstances. Recent work with Syrian refugee therapists, highlighted higher levels of secondary traumatic stress in professional quality of life assessments when compared to other populations [41].

Furthermore, what is deemed ‘acceptable politics’ within mental health spaces, is influenced by norms within the wider public sphere (hegemonic domains of power) and the views of those who have access to forms of power to drive and shape the actions and practices of others. For example, shifting discourses create windows of acceptability for expressing solidarity with certain groups. In the first half of the 2020’s, Black Lives Matter movements gained increased acceptability and support in public domains, in ways that rarely acknowledged this as connected to a legacy of Black liberation struggles and was eventually met with systemic rejection when these links were made explicit by activists [42]. In some spaces, expressing solidarity for issues such as abortion rights or the Palestinian fight for self-determination are validated, while in others it is labeled outside the bounds of ‘acceptable’ politics, often within in the same country.

As such, organisational responses to ‘political’ issues are always shaped by broader societal climates. Recognition of these realities does not automatically mean that an organisation has embedded social justice concerns into policies and practices, leaving them psychologically unsafe for those in minoritised bodies. This speaks to environmental preparedness and organisations’ ability or willingness to centre safety for all – recognising no space can be truly safe when power hierarchies prevail – rather than prioritising safety of those with powerful voices and resources. Further, the intergenerational exposure and cumulative toll of this structural blindness perpetuates community distrust and differential treatment outcomes [43]. Communities are hit with the direct impacts of oppression, but also the burden of advocating for their own humanity and safety. Even within mental health spaces these communities often cannot safely name or address the political realities impacting their mental health, which not only limits treatment effectiveness but recreates the very -traumatising conditions of silencing and invalidation that contribute to psychological harm.

Intersectionality drives us to resist structural blindness in our response, by forcing us to acknowledge the work that social domains and structures do to establish specific identities – which are themselves defined by their own articulations and sense making within social realities [44]. With an intersectional socio-political lens, it becomes impossible to work with and support the good mental health of individuals without asking the question: *what is it about your world that makes you ill*, and beyond that – *what can we do about this?* An intersectional approach to therapeutic and care practices acknowledges that social realities are not additive – but constitutive of a specific experience that cannot be addressed without action in the structural domains of people’s lives. Intersectionality, combined with a socio-political economy of social determination –also brings an articulation of power as it operates at the macro, meso, family and kin levels, into how we address mental health needs – ultimately drawing into focus the fact that mental health equity cannot be achieved without efforts to address the historical, social and political landscapes that determine people’s access to the resources that enable or hinder mental health improvements. The framework seeks to foreground the interdependent, dynamic interactions that produce variations in mental health inequalities across different communities and contexts. Crucially, centralising the mechanisms of discrimination, exclusion, and oppression calls attention to *why* individuals and communities who hold minoritised and marginalised identities experience inequalities – not just that they do. This distinction is critical: identities are not risk factors - differential treatment, experiences, and inequitable systems faced by some, are the actual drivers of poor mental health outcomes.

Responding to these challenges in practice is supported by explicit frameworks that can prompt reflection on the mechanisms through which structural oppression operates, providing a foundation for justice-oriented policy and practice that acknowledges root causes and contextual realities in producing differential mental health outcomes across communities and contexts. Below we outline our approach which contributes to this space.

## 2 Introducing a socio-political economy of global mental health: Domains of social determination

Following a series of discussions by the authors, alongside a review of key socio-political economic literature [15,34,45,46], we proposed an initial framework for a Socio-Politicalal Mentalb Economy of Global Health to expand and extend our analyses and actions in the global mental health field (see [2]). We identified nine social phenomena which are well articulated within social theory as contributing to the emergence, patterning, and embeddedness of commonly cited social determinants of mental health: (1) Political Dynamics, (2) Racism, Caste & Xenophobia, (3) Gender & Sexuality, (4) Neighbourhood Dynamics, (5) Class & Working conditions, (6) Colonialism, (7) Indigeneity (8) Religious & Spiritual Identities (9) Age & Disability (see [47,48]). Underpinning the selection of these domains, is an interest in centering social mechanisms of oppression and exclusion; which are the processes through which the interactions between social actors, places and systems are shaped, as discussed in earlier sections of this essay.

Through the explicit labelling of these domains, we seek to center specific social conditions and dynamics that are often underexplored, or overlooked within the global mental health landscape, providing a starting point for more structurally-informed formulations of action for mental health improvements, rather than a prescriptive template. These domains are examples of the social factors that appear within the general domain described by Briehl [35], and give us an opportunity to grapple with and hold the complexity linked to the social determination of mental health. Further justification of these decisions are described in our earlier papers (see [2]). Crucially however, these domains do not present a comprehensive checklist – and all attempts to apply this framework in new settings would require practitioners and actors to reflect on the relative importance of each domain, and specific aspects within it, in making sense of unique socio-political-historical contexts. In the remainder of this section, we outline the broad definitions of each domain, and their potential relationship to poor mental health outcomes. A table summarizing these dynamics can be found in our previous review [2].

***Political Dynamics*** encompasses both de jure and de facto practices related to government policy-making, legislation, regulation of people, borders, residency, work, healthcare, as well as access to safe sanctuary and protection. Inclusive of political climate and contextually relative processes of minoritisation, understanding both minority and majority contexts. For example, reproductive rights restrictions following the overturn of the constitutional right to abortion in the US in 2022 led to significant mental health deterioration across the population, particularly among those with low income and low education levels [49]. Similarly, hostile immigration policy reforms in England negatively impacted mental health among Black Caribbean communities, reflecting broader colonial legacies recreated in contemporary policy [50].

***Racism, Caste & Xenophobia*** includes factors related to any distinction, exclusion, restriction preference or antagonism of a person or people on the basis of their skin colour, hair texture, ancestral origin, nationality, or membership of a particular racial or ethnic group, typically minoritised or marginalized. This includes the denial of equal rights or exclusion based on an individual or groups’ geographic origins or values, beliefs and/or practices that are perceived as foreign or originating from outside a community or nation. It also includes social stratification systems and practices based on beliefs of hereditary transmission of a style of life, determined by birth and remains fixed for life and exclusion based on cultural notions of purity and pollution. For example, the mental health implications of caste-based discrimination and exclusion experienced by Dalit women in India [51] or the experiences of rural migrant workers in China [52].

***Gender Identity & Sexuality*** encompasses factors related to social constructs of norms, behaviors, and roles, as well as prejudice, stereotyping, or discrimination based on assigned sex, gender identity, and/or sexual orientation. This includes attitudes, bias, and discrimination that favor heteronormative relationships and the belief that heterosexuality is the only “normal” sexual orientation. For example, binary gender norms combined with the criminalisation of homosexuality through the Same-Sex Marriage (Prohibition) Act 2013 significantly worsens mental health outcomes for LGBT+ communities in Nigeria [53].

***Neighbourhood Dynamics*** refers to attitudes, behaviors, and systems of policies toward housing, shelter, or settlement constructed on land without legal claim, falls outside government regulation, or is not afforded state protection or in compliance with current planning and building regulations. This includes residential areas often stigmatised or associated with poverty, those consisting of densely packed housing units of weak build quality, as well as processes of ghettoization or displacement where low-income residents are forced from their neighborhoods due to influxes of wealthier residents and increasing property values. These dynamics create concentrated disadvantage that compounds mental health risks through environmental stressors, social isolation, and reduced access to resources. For example, the mental health impacts of living in an area segregated by a ‘peaceline’ between Catholics and Protestants in Northern Ireland [54], structural conditions of Congolese refugees living in refugee camps in Uganda and Rwanda [55] or exposure to local crime, drug selling, poor sanitation and local economic disadvantage in poor urban communities in Accra [56].

***Class & Working Conditions*** encompasses classism and individual attitudes, behaviours, systems of policies and practices designed to benefit the upper/dominant classes at the expense of the lower/subordinated classes, including beliefs that a person’s social or economic station in society determines their societal value. This domain also addresses attitudes, behaviours, systems of policies and practices towards conditions of work which influence the likelihood of workplace accidents, injuries and diseases as well as economic activities that have market value but are not formally registered or insufficiently covered by formal arrangements. Failures to eliminate child labour and the subsequent mental health impacts in Indonesia [57] or class-based obstacles experienced by working class academics contributing to mental health challenges in the UK higher education landscape [58], are two exaples.

***Colonialism*** captures factors related to the domination of peoples or areas by foreign states or nations including practices of extending and maintaining a nation’s political and economic control over another people or area as well as the replacement of indigenous populations with an invasive settler society that, over time, develops a distinctive identity and sovereignty claims over colonised territories. Examples include research exploring the impact of colonial mentality and expectations around acculturation on Pilipinx Americans [59] or historical trauma due to colonisation on Indigenous young people’s mental health [60].

***Indigeneity*** *a*ddresses factors affecting populations who self-identify as original peoples of the land who were displaced and marginalised by colonisers or later settlers, as well as efforts to reclaim and revive Indigenous identities and cultural practices, regain or retain land rights, human rights, heritage, and political standing. The mental health impacts on Indigenous communities who experience land dispossession due to mining, hydroelectric dams, petroleum, and agricultural developments across Australia, Aotearoa, North and South America, and the Circumpolar North are well established [61] or the role of Indigenous cultural identity as protective against poor mental health [62].

***Religious & Spiritual Identities*** encompasses discrimination, fear, hatred, or prejudice against religious or spiritual belief systems, including denial of people’s rights to practice and express their beliefs freely as well as discrimination performed by a religious group or using spiritual texts to justify harms to another. For example, the political violence, genocide and mass displacement of the Rohingya people based on their ethnic and religious identity has had significant mental health implications [63] or research exploring the mental health impacts of religion-based LGBTQA+ discrimination [64].

***Age & Disability*** addresses discrimination, unfair treatment, and social prejudice against people with physical or mental disabilities or differences, as well as discrimination and stereotyping based on physical age. This domain intersects significantly with other factors, as seen in discrimination based on HIV status, which has consistently been associated with poor mental health [65,66], is classified as a disability under both the UK Equality Act and the US Americans with Disabilities Act. However, many countries do not classify HIV as a disability under their laws, demonstrating how health conditions become sites of multiple, intersecting oppressions. Further research on ageism intersecting with sexuality-related stigma [67] or subjective social status [68] indicates negative impacts on psychological distress and body-based stress cortisol.

Finally, we acknowledge space for ‘*Other*’ domains to acknowledge this as an ongoing learning and unlearning process. In our review [2], we identified additional factors such as language and family dynamics that may require separate consideration in specific contexts, underscoring the framework’s need for contextual adaptation and expansion. In order to acknowledge the complexity of social worlds, a framework should always leave space and flexibility for the fact that manifestations of oppression are continually evolving.

## 3 The socio-political economy of global mental health framework: Creating spaces to visualise health equity and justice

By articulating an intersectional approach to the socio-political economy of mental health, this framework directs actors to hold onto the complexity of people’s experiences in an unjust world, to enable awareness of, and conversations across differing levels of power - the interpersonal, structural, disciplinary and hegemonic domains - at work in people’s lives [8,69]. Our framework depicts how structural determinants work as factors of oppression creating continual interactions, amplifying unique experiences of risk of poor mental health among individuals and communities. As such, this offers a tool that can be used to frame efforts to work towards mental health equity and promote critical reflection on the ways organisational practices are in service of or may be failing certain communities by revealing how seemingly neutral policies reproduce patterns of exclusion. Moving beyond understanding the impact of discrimination and oppression as risk factors for mental ill-health has implications for interventions, as it directs us to potential actions that could work to prevent further harm and advance health equity at multiple domains. This brings into consciousness the role of mental health professionals, organisations and commissioning bodies, locating their efforts as either operating to treat individual pathology alone, or as part of broader social justice efforts to dismantle oppressive systems.

To conclude this essay we reflect on ways the framework can shift transformative action across three interconnected levels: systems of care, intervention approaches, and policy environments. Rather than treating these as separate domains, the framework reveals how changes at each level must work together to address the structural roots of mental health inequities.

### 3.1 Creating new logics of care: A caring mental health system

Creating caring mental health systems requires embedding anti-oppression and cultural safety at all levels. For this to be possible, requires a shift in how ‘care’ within ‘mental health care’ is itself, conceptualised. Burgess [8] argues that care systems need to be oriented towards logics of ‘remedy and repair,’ which are defined as the need to make right the ruptures in social worlds that are the foundations of distress, and that establish the patterning of illness. This is rooted in the acceptance that mental health care, defined by those who use it, may not always look like psychological support - instead embody quests for justice. This perspective draws on activist and anthropological orientations to care. For example, anthropological studies of care direct us to the ways in which care can be understood more broadly, to include a focus on sociopolitical and geopolitical landscapes that articulate the need for change in our practices of support for each other and our relationships to the world [70]. Activist orientations to care, build on this idea of care as expanding beyond typical binary medicalized approaches (illness and treatment) to articulate it as a purposefully political act. As such, mental health systems oriented towards remedy and repair would include robust pathways for accessing privileges associated with human rights and dignity across multiple domains, including education, welfare, as well as health services.

Because individuals are embedded within systems, interventions cannot exist free of the structural contexts and realities in which people live. Care as repair embraces the fact that people’s desires to access economic opportunity, tackle racism, violence, sexism, and establish safety in their lives, as neccessary care acts - and neccessary parts of services. The value of complex approaches to care that enable recognition of multiple dimensions of social life is immeasurable. Guilaine Kinouani [71] also discusses the importance of therapeutic interventions and treatments moving towards interests in our view of social justice, which demands a sensitivity and willingness for practitioners to engage in work that actively recognizes the harms of racism(s), exclusions, and the impact of systems on mental health. This closely aligns with Cristina Sharpe’s articulations of care as political work – that is attuned to the ongoing violence that many marginalised and oppressed bodies face, historically, and in the present [72].

Some may feel that community-based interventions provide a route to caring mental health systems. However, engagement with communities, does not automatically demand the acknowledgement or prioritisation of community desires, nor parallel political work to change the realities of those based in fractured communities. And while some methodologies - such as co-design and coproduction - bring us closer to listening to communities, these interventions are not always transformative in their aims or desires for wider community worlds, viewing community engagement as a means to achieve a goal, rather than a process or site for the transformation of their broken social worlds [73].

Our framework offers an alternative starting point to anchor community engagement with context to history, contemporary needs, and power dynamics in any new setting. For example, rather than treating structural factors as separate or passive background considerations, therapeutic interventions informed by our framework would explicitly explore how Political Dynamics and exposure to political climate and legislation intersects with a service user’s religious (*Religion & Spirituality*) belief systems or *Gender & Sexuality* in their experience, This aligns with calls to expand competency frameworks to attune to complex systems where people live [74]. For example, in their current form, many peer-support programmes revolve around illness identities alone. Such approaches fail to recognize the intersectional complexity of those living with mental ill health conditions, often excluding those who may feel additional positionalities liked to class, caste, race, or other categories, shape their experiences of distress more than the condition itself. The result is a simplification that limits the wider value of such interventions in complex communities. The application of our framework would ensure that design of peer-support programmes mapped the complexity of identifies as they present in particular contexts, exploring what mental health conditions mean to them, and how other domains of being also shape access to care and treatment and support from peers.

We also anticipate that our framework could also be valuable for shaping approaches to recruitment in clinical trials and organising implementation science efforts more broadly. The framework supports the inclusion of communities that are typically excluded, by resisting the idea of the ‘ideal patient’. Instead our framework pushes researchers to intentionally design recruitment strategies that include those with intersecting vulnerabilities and complex identities, ensuring that trial samples capture a wider range of experiences. With respect to implementation science, our framework could be applied to evaluation frameworks, such that standard constructs typically used to understand implementation process (i.e., acceptability, feasibility) explicitly consider the complexity of people’s lives who would utilise interventions in real world settings. As a result, we could develop better understandings of how delivery may unintentionally reproduce exclusions, where assumptions have been made about cultural appropriateness in a decontextualised manner.

Building caring health systems may also look like service level actions which model themselves after activist-care systems. Early work by Susan Hagedorn [75] defined activist oriented primary care as a process that promotes activities of shifting the social power-dynamics that anchors people and communities in states of disease, alongside activities of providing individual care. This aligns with longstanding traditions of social and collective medicine from numerous Latin American countries, which are driven by the need to address social determination of poor health [76]. Specifically, Breilh describes critical interculturality as a process that moves us beyond an awareness of power, and to exploring how we work with power to shift practices that redress societal patterns of risk and access leading to poor health outcomes.

How might we take critical interculturality into practice in caring mental health systems? One route may include championing cultural safety [77]. This approach focuses on creating an environment where everyone feels respected and understood, regardless of their cultural background. The paradigm emerged as a critique of mainstream health practices and engagements with Māori and western pacific indigenous communities in the 1990’s. Over time, perspectives were expanded by Indigenous scholarship and allied health practitioners globally, as part of efforts to address and understand the way systems and practices work in ways that ultimately keep people ‘unsafe’. The practice is oriented towards embodiment of reflexivity that engages with the wider structural domains that drive health system failures. It demands an acceptance of power, history, and inequities experienced systemically, and rejects the assumption that systems in their existing forms are safe for everyone. In the US, radical social work scholars have also articulated the power of simple frameworks to operationalize thinking about these issues within care practices. Belkin Martinez and colleagues’ [78] work on the Liberation Health Framework articulates that individuals must be situated within three contexts prior to determining their care needs: Institutional factors, Cultural factors, and Personal factors. By using this framework alongside tools like the liberation health framework or cultural safety toolkits, we are able to define and name the harmful factors, and through naming them think through how they can be held within care through actionable steps, that stage short and long-term strategies for mental health improvement.

Ultimately, the creation of a caring mental health system is a political process; as the design, organisation, curation and funding of mental health systems, are politically determined. Our model illuminates some of the domains where political actions, or the presence or absence of political will, establish the conditions through with poor mental health is established, and lived. Building a caring mental health system moves us to a place where services are situated within the landscape of wider political action and decision making, particularly in non-health sectors. Caring systems require removing threat and policing by commissioning bodies to enable organisations to work with communities in community-led ways. This requires more robust systems of accountability, alongside enabling policy environments at organisational level acknowledging the capacity of systems to operate as sites of discrimination. That is, a remedy and repair-oriented system would systematically address how each domain in our framework creates barriers to care.

For example, when *Political Dynamics* criminalise or prime reactivity to certain identities or belief systems, repair-oriented systems must act intersectionaly. For example, within the mental health to prison pipeline, which in the UK has been linked to the misuse of diagnostic categories by criminal justices structures, such as cannabis psychosis, in the 1980s, which led to arrests of primarily young black men from Jamaican backgrounds [79]. A caring system would provide sanctuary, rather than compliance with harmful policies, and articulate how criminialisation of certain actors occurs. This might include mental health services that also provide legal advocacy alongside therapy when clients face immigration threats, or wider campaigns to work to decriminalise deaths by suicide, in countries where crimianlisation of these events limits people seeking help around in context of failed attempts on one’s life, and wider stigma towards affected individuals [80]. When *Colonialism* shapes organisational hierarchies, care as repair means decolonising decision-making processes and centering Indigenous ways of healing. This would look like healing spaces that, for example explicitly acknowledge historical trauma from residential schools but integrate advocacy for the realisation of truth and reconciliation acts, in Canada. Implementing these approaches requires specific organisational changes: staff training, policies that explicitly name and address discrimination within services, partnerships with community advocacy groups, and funding structures that support long-term relationship-building rather than short-term individual treatments.

A caring system is also one that responds to the living nature of society and the need for social action in creating the possibilities for health. It acknowledges that this is not static work, but calls for vigilance to the way vectors of inequality shift through time. The system must be built with sufficient flexibility that it can acknowledge both the enduring impact of the past as well as the ways the past is re-born in the present. – For example, resurgences of hyper-vigilant and violent forms of masculinity, alongside the erosion of policy and legislative protections for marginalised peoples. These examples remind us that the political domain, as with others, is always a live space; that they are constantly produced within temporal and social contexts, continually shaped by overt and covert political discourse, wider political climates, and associated power relationships. A health system that fails to respond to these dynamics will never be safe.

Contributions towards struggles for liberation of marginalised groups, and widescale education on their collective dimension must be seen as part of building mental health enabling societies and countries [81]. With this in mind, application of a socio-political economic framework guides us more accurately towards specific political domains, including harmful policies that carry mental health consequences – such as the literature detailing xenophobic political landscapes established pathways to poor mental health outcomes. Just as we implement sugar taxes to address dietary health risks, we need equivalent structural interventions targeting the commercial and political determinants of mental health inequities. Recent class-action suits against tech companies linked to young people’s mental health begin to pave the way for large scale political action. This draws attention to the need not only for specific mental health interventions for those affected by these policies, but also for exploring how mental health promoting policies must become woven into everyday fabric of social life. For example, within engagement with policy makers in ministries of health, the framework could be used to highlight the links of mental health inequalities to the political economy of poor health in general – making space to discuss how policy recommendations embrace structural reforms alongside individual treatment expansion. Examples of promising approaches where the framework could build on existing interests in complexity, include Scotland’s integration of human rights into legislation, Wales’s strategy of integrating mental health concerns into all policies, and recent WHO updated guidance on mental health action plans which similarly call mental health mainstreaming across government sectors [82,83].

## 4 Conclusion

In this essay, our main objective was to illuminate the value of our framework for a socio-political economy of mental health, grounded in social determination and intersectionality, to efforts to build mental health interventions, services and systems that expand visions of justice towards transformation. We argue that using such a lens may guide policy approaches to mental health promotion and prevention rooted in the complexities of social landscapes; and the development and delivery of treatment and community support in ways that acknowledge intersecting forms of oppression and discrimination that should always be considered within social determinants approaches.

This framework is particularly important in the space of Global Mental Health, as it guides a pathway to build on the successes of a social determinants approach which established the necessity of the social for better mental health for many. However, this essay underscores the frameworks value in a truly global sense, with clear importance in high and low resource settings alike. The Socio Political Economy of global mental health framework articulates a shift from the necessary to the sufficient in mental health, which takes seriously, the social, structural, cultural, historical and economic processes that drive manifestations and patterning of poor mental health. We hope that it will guide treatment and policy actors in our field to embrace and lead action against the more complex social and political systems that determine our mental health, in all places, and all times.

## References

[1] Riley CA. Black liturgies: prayers, poems, and meditations for staying human. New York: Convergent Books. 2024.

[2] Burgess RA, Chobhthaigh SN, Biswal B, Ceccolini D, Fadipe B, Khan D, et al. Intersectional discrimination, exclusion and the socio-political economy of global mental health: A systematic scoping review of the literature. SSM - Mental Health. 2025;7:100382. doi: 10.1016/j.ssmmh.2024.100382

[3] Foucault M. The subject and power. In: Power: the essential works of Foucault 1954–1984. London: Penguin Books. 2002. 326–48.

[4] Ingleby D. Critical psychiatry: the politics of mental health. London: Free Association Press. 1980.

[5] Fanon F. Alienation and freedom. London: Bloomsbury Publishing. 1954/2022.

[6] Rimke H. Introduction – Mental and Emotional Distress as a Social Justice Issue: Beyond Psychocentrism. SSJ. 2016;10(1):4–17. doi: 10.26522/ssj.v10i1.1407

[7] Thrift E, Sugarman J. What is social justice? Implications for psychology. J Theor Philos Psychol. 2019;39(1):1.

[8] Burgess RA. Rethinking global health: frameworks of power. 1st ed. London: Routledge; 2023. doi: 10.4324/9781315623788

[9] Kirkbride JB, Anglin DM, Colman I, Dykxhoorn J, Jones PB, Patalay P, et al. The social determinants of mental health and disorder: evidence, prevention and recommendations. World Psychiatry. 2024;23(1):58–90. doi: 10.1002/wps.21160 38214615 PMC10786006

[10] Fraser N. Scales of justice: reimagining political space in a globalizing world. New York: Columbia University Press. 2009.

[11] Sankara T. We are the heirs of the world’s revolutions: speeches from the Burkina Faso revolution 1983–1987. Atlanta: Pathfinder Press. 2007.

[12] Young IM. Justice and the politics of difference. Rev ed. Princeton (NJ): Princeton University Press. 2011. doi: 10.2307/j.ctvcm4g4q

[13] Sugarman J. Neoliberalism and psychological ethics. J Theor Philos Psychol. 2015;35(2):103–16.

[14] Ridley M, Rao G, Schilbach F, Patel V. Poverty, depression, and anxiety: Causal evidence and mechanisms. Science. 2020;370(6522):eaay0214. doi: 10.1126/science.aay0214 33303583

[15] Burns JK. Poverty, inequality and a political economy of mental health. Epidemiol Psychiatr Sci. 2015;24(2):107–13. doi: 10.1017/S2045796015000086 25746820 PMC6998107

[16] Carrillo B, Charris C, Iglesias W. Moved to Poverty? A Legacy of the Apartheid Experiment in South Africa. American Economic Journal: Economic Policy. 2023;15(4):183–221. doi: 10.1257/pol.20210439

[17] Jewkes R, Milovanovic M, Otwombe K, Chirwa E, Hlongwane K, Hill N, et al. Intersections of sex work, mental ill-health, IPV and violence among female sex workers in South Africa. Int J Environ Res Public Health. 2021;18(22):11971. doi: 10.3390/ijerph182211971 34831727 PMC8620578

[18] Kerr P, Durrheim K, Dixon J. Beyond the two-group paradigm in intergroup conflict and inequality. Br J Soc Psychol. 2017;56(1):47–63. doi: 10.1111/bjso.12163 27888516

[19] Badgett MVL, Choi SK, Wilson BDM. LGBT poverty in the United States. Los Angeles (CA): Williams Institute, UCLA. 2019. https://williamsinstitute.law.ucla.edu/wp-content/uploads/National-LGBT-Poverty-Oct-2019.pdf

[20] Lawrence PR, Anderson RK. Poverty and Disability: A State-Level Geospatial Analysis. Clin Nurs Res. 2024;33(5):344–54. doi: 10.1177/10547738241249834 38770759

[21] The Lancet Public Health. Poverty is a political choice. Lancet Public Health. 2018;3(12):e555. doi: 10.1016/S2468-2667(18)30243-3 30473484

[22] Shiva V. Seeds of suicide: the ecological and human costs of seed monopolies and globalisation of agriculture. New Delhi: Navdanya. 2006. https://sanhati.com/wp-content/uploads/2007/08/seeds-of-suicide.pdf

[23] Kannuri NK, Jadhav S. Cultivating distress: cotton, caste and farmer suicides in India. Anthropol Med. 2021;28(4):558–75. doi: 10.1080/13648470.2021.1993630 34730036 PMC8734467

[24] Ideal Homes. ND A history of South East London Suburbs: A history of Brixton. https://ideal-homes.gre.ac.uk/lambeth/lambeth-assets/histories/brixton.html

[25] Watt P. Taking a long view perspective on estate regeneration: before, during and after the New Deal for Communities in London. J Hous and the Built Environ. 2023;38:141–70. doi: 10.1007/s10901-022-09929-1

[26] Perrera J. The London Clearances: Race, Housing and Policing. Institute of Race Relations. 2019. https://irr.org.uk/wp-content/uploads/2020/09/The-London-Clearances-Race-Housing-and-Policing-1.pdf

[27] Niessen S. Shattering the Silence: The Hidden History of Indian Residential Schools in Saskatechewan. https://collections.irshdc.ubc.ca/media/collectiveaccess/images/1/2/5/11575_ca_object_representations_media_12535_original.pdf

[28] Janes K, Vernon P, Estefan D, Sheibani F, Caesar G, Burgess RA. The ties that bind: mental health consequences of the Windrush scandal. SSM Ment Health. 2024;6:100352.

[29] Mignot JF. Decriminalizing homosexuality: a global overview since the 18th century. Ann Demogr Hist. 2022;143(1):115–33. doi: 10.3917/adh.143.0115

[30] International Court of Justice. Application of the Convention on the Prevention and Punishment of the Crime of Genocide in the Gaza Strip (South Africa v Israel). 2024. https://www.icj-cij.org/node/204091

[31] Burgess RA. The struggle for the social: rejecting the false separation of “social” worlds in mental health spaces. Soc Psychiatry Psychiatr Epidemiol. 2024;59(3):409–16. doi: 10.1007/s00127-023-02510-3 37400665 PMC10944383

[32] Krieger N. Discrimination and health inequities. Int J Health Serv. 2014;44(4):643–710. doi: 10.2190/HS.44.4.b 25626224

[33] Schrecker T. The Political Economy of Public Health: Challenges for Ethics. The Oxford Handbook of Public Health Ethics. Oxford University Press. 2019. 841–56. doi: 10.1093/oxfordhb/9780190245191.013.73

[34] Glasgow S, Schrecker T. The double burden of neoliberalism? Health Place. 2016;39:204–11. doi: 10.1016/j.healthplace.2016.04.003 27132687

[35] Breilh J. Critical epidemiology and the people’s health. Oxford: Oxford University Press. 2021.

[36] Harvey M, Piñones-Rivera C, Holmes SM. Thinking with and against the social determinants of health. Int J Health Serv. 2022;52(4):433–41. 36052418 10.1177/00207314221122657

[37] Faissner M, Gaillard A-S, Juckel G, Yeboah A, Gather J. Intersectionality and discriminatory practices within mentalhealth care. Philos Ethics Humanit Med. 2024;19(1):9. doi: 10.1186/s13010-024-00159-7 38937818 PMC11212199

[38] Thornicroft G, Sunkel C, Aliev AA, Baker S, Brohan E, El Chammay R, et al. Ending stigma and discrimination in mental health. Lancet. 2022;400(10361):1438–80. doi: 10.1016/S0140-6736(22)01470-2 36223799

[39] Das R. Identity politics: a Marxist view. Class Race Corp Power. 2020;8(1).

[40] Castaldelli-Maia JM, Bhugra D. What is geopsychiatry? Int Rev Psychiatry. 2022;34(1):1–2. doi: 10.1080/09540261.2022.2031915 35584019

[41] Hamid A, Scior K, Abdul-Hamid W, Williams ACDC. Displaced Syrian mental health workers. J Refug Stud. 2021;34(2):2394–405. doi: 10.1093/jrs/feaa068

[42] Khan-Cullors P, Bandele A. When they call you a terrorist: a Black Lives Matter memoir. New York: Macmillan. 2018.

[43] Price TJ, McGowan VJ. A cycle of social violence. Soc Sci Med. 2025;118438. 40695055 10.1016/j.socscimed.2025.118438 PMC7619319

[44] Butler C. Intersectionality and systemic therapy. Context. 2017;(151):16–8.

[45] True J. The political economy of violence against women. Oxford: Oxford University Press. 2012.

[46] Young J, McGrath R. Exploring discourses of equity and social justice. Aust J Prim Health. 2011;17(4):369–77. 22112706 10.1071/PY11038

[47] Lund C, Brooke-Sumner C, Baingana F, Baron EC, Breuer E, Chandra P, et al. Social determinants of mental disorders. Lancet Psychiatry. 2018;5(4):357–69. doi: 10.1016/S2215-0366(18)30060-9 29580610

[48] World Health Organization. World mental health report: transforming mental health for all. Geneva: WHO. 2022.

[49] Anderson MR, Burtch G, Greenwood BN. The impact of abortion restrictions on American mental health. Sci Adv. 2024;10(27):eadl5743. doi: 10.1126/sciadv.adl5743 38959323 PMC11221505

[50] Jeffery A, Gascoigne C, Dykxhoorn J, Blangiardo M, Geneletti S, Baio G, Kirkbride JB. Effect of immigration policy reform on mental health in people from minoritised ethnic groups in England. Lancet Psychiatry. 2024;11(3):183–92. doi: 10.1016/S2215-0366(23)00412-1 38360023 PMC11101585

[51] Rani A, Raman KJ, Antony S, Thirumoorthy A, Chethan B. Microaggression and Its Impact on Lower Caste Women with Psychiatric Disorders in India. Violence and Gender. 2025;12(1):35–42. doi: 10.1089/vio.2024.0041

[52] Liu S, Zhang K, Ren Q. A dynamic relationship between discrimination and psychological distress among rural migrant workers in China. Chinese Journal of Sociology. 2024;10(4):563–84. doi: 10.1177/2057150x241289704

[53] Ogunbajo A, Iwuagwu S, Williams R, Biello KB, Kahler CW, Sandfort TGM, Mimiaga MJ. Experiences of minority stress among GBMSM in Nigeria. Glob Public Health. 2021;16(11):1696–710. doi: 10.1080/17441692.2020.1834598 33108249 PMC8076332

[54] Maguire A, French D, O’Reilly D. Residential segregation, dividing walls and mental health. J Epidemiol Community Health. 2016;70(9):845–54. 26858342 10.1136/jech-2015-206888 PMC5013154

[55] Chiumento A, Rutayisire T, Sarabwe E, et al. Mental health problems of Congolese refugees in Rwanda and Uganda. Confl Health. 2020;14:77. doi: 10.1186/s13031-020-00323-8 33292363 PMC7670672

[56] Greif MJ, Dodoo FN. Community stressors and mental health in urban slums of Accra. Health Place. 2015;33:57–66. 25754264 10.1016/j.healthplace.2015.02.002

[57] Jayawardana D, Baryshnikova NV, Cheng TC. Long shadow of child labour on adolescent mental health. Empir Econ. 2023;64:77–97. doi: 10.1007/s00181-022-02241-5

[58] Crew TF. Working-class academics and mental health. SN Soc Sci. 2025;5(5):66.

[59] Sevillano L, Paez JM, Covington E. Restoring *kapwa*: colonial mentality among Pilipinx Americans. Asian Am J Psychol. 2023;14(4):307–26. doi: 10.1037/aap0000314

[60] Smallwood R, Woods C, Power T, Usher K. Historical trauma and Indigenous youth well-being. J Transcult Nurs. 2021;32(1):59–68. doi: 10.1177/1043659620935955 32567510

[61] Ninomiya ME, Burns N, Pollock NJ, Green NT, Martin J, Linton J, et al. Mental health impacts of land dispossession in Indigenous communities. Lancet Planet Health. 2023;7(6):e501–17. 10.1016/S2542-5196(23)00079-7 37286247

[62] Walker S, Kannan P, Bhawra J, Katapally TR. Digital citizen science and Indigenous youth mental health. PLoS One. 2023;18(12):e0294234. 10.1371/journal.pone.0294234 PMC10735025 38127846

[63] Khan NZ, Shilpi AB, Sultana R, Sarker S, Razia S, Roy B, et al. Mental health risks among displaced Rohingya children. Child Care Health Dev. 2019;45(1):28–35. 30335204 10.1111/cch.12623

[64] Jones TW, Power J, Jones TM. Religious trauma and moral injury from LGBTQA+ conversion practices. Soc Sci Med. 2022;305:115040. doi: 10.1016/j.socscimed.2022.115040 35609469

[65] Armoon B, Fleury MJ, Bayat AH. HIV-related stigma and mental health outcomes. Int J Ment Health Syst. 2022;16:17. doi: 10.1186/s13033-022-00527-w 35246211 PMC8896327

[66] Rueda S, Mitra S, Chen S, Gogolishvili D, Globerman J, Chambers L, et al. HIV-related stigma and health outcomes. BMJ Open. 2016;6(7):e011453. 10.1136/bmjopen-2016-011453 PMC4947735 27412106

[67] Lyons A, Alba B, Waling A, Minichiello V, Hughes M, Barrett C, et al. Ageism, sexuality-related stigma and mental health. Aging Ment Health. 2022;26(7):1460–69. doi: 10.1080/13607863.2021.1978927 34528497

[68] Weiss D, Weiss M. Subjective social status and cognitive aging beliefs. Psychophysiology. 2016;53(8):1256–62. 27159187 10.1111/psyp.12667 PMC4949085

[69] Collins PH. Black feminist thought: knowledge, consciousness, and the politics of empowerment. 2nd ed. New York: Routledge; 2022.

[70] Drotbohm H. Care Beyond Repair. Oxford Research Encyclopedia of Anthropology. Oxford University PressNew York: NY. 2022. doi: 10.1093/acrefore/9780190854584.013.411

[71] Kinouani G. Living while Black: the essential guide to overcoming racial trauma. London: Random House. 2021.

[72] Sharpe C. In the wake: on blackness and being. Durham (NC): Duke University Press. 2016. doi: 10.1215/9780822373452

[73] Williams O, Sarre S, Papoulias SC, Knowles S, Robert G, Beresford P, et al. Lost in the shadows: reflections on the dark side of co-production. Health Res Policy Syst. 2020;18(1):43. doi: 10.1186/s12961-020-00558-0 32380998 PMC7204208

[74] Beagan BL. Critique of cultural competence. In: Frisby C, O’Donohue W. Cultural competence in applied psychology. Cham: Springer. 2018. 1–24. doi: 10.1007/978-3-319-78997-2_6

[75] Hagedorn S. Politics of caring: activism in primary care. Adv Nurs Sci. 1995;17(4):1–11. 10.1097/00012272-199506000-00002 7625777

[76] Pentecost M, Adams V, Baru R, Caduff C, Greene JA, Hansen H, et al. Revitalising global social medicine. Lancet. 2021;398(10300):573–4. doi: 10.1016/S0140-6736(21)01003-5 34058131

[77] Curtis E, Jones R, Tipene-Leach D, Walker C, Loring B, Paine S-J, et al. Why cultural safety rather than cultural competency is required to achieve health equity: a literature review and recommended definition. Int J Equity Health. 2019;18(1):174. doi: 10.1186/s12939-019-1082-3 31727076 PMC6857221

[78] Belkin Martinez D, Fleck-Henderson A. Social justice in clinical practice: a liberation health framework for social work. London: Routledge. 2014.

[79] Fernando S. Mental health, Race and Culture (3rd Edition). London: Bloomsbury Press. 2008.

[80] Oyetunji TP, Arafat SY, Famori SO, Akinboyewa TB, Afolami M, Ajayi MF, et al. Suicide in Nigeria: newspaper content analysis. Gen Psychiatry. 2021;34(1):e100347. 10.1136/gpsych-2020-100347 PMC7812081 33521558

[81] Pathare S, Burgess RA, Collins PY. World Mental Health Day: prioritise social justice. Lancet. 2021;398(10314):1859–60. doi: 10.1016/S0140-6736(21)02232-7 34627493

[82] Welsh Parliament. Mental health strategy for Wales. Cardiff: Welsh Government. 2022.

[83] World Health Organization. Guidance on policy and strategic actions to protect and promote mental health across government sectors. Geneva: WHO. 2025. https://www.who.int/publications/i/item/9789240114388

